# Virtual Geriatric Education Preferences of Rural Social Workers

**DOI:** 10.1093/geroni/igab046.1267

**Published:** 2021-12-17

**Authors:** Joan Ilardo, Raza Haque, Angela Zell

**Affiliations:** 1 Michigan State University College of Human Medicine, East Lansing, Michigan, United States; 2 Michigan State University, East Lansing, Michigan, United States

## Abstract

Older adults in rural communities need access to comprehensive healthcare services provided by practitioners equipped with geriatric knowledge and skills. The Geriatric Rural Extension of Expertise through Telegeriatric Service (GREETS) project goal is to use telemedicine and telehealth to expand geriatric service options to underserved Michigan regions. GREETS educational programs train health practitioners to provide geriatric care for vulnerable older adults. To determine gaps in geriatric competencies, the team conducted an online survey of health professionals including behavioral health practitioners. Respondents identified educational topics and preferred virtual delivery methods. Demographic information included respondent’s professional position, practice setting, and county. The respondents were asked to indicate level of educational need using a scale ranging from a low, medium, or high need. Fifty (47%) of 106 total responses were from social workers. We compared the percent of social workers to other practitioners’ responses in our analysis. Four topics emerged for both groups as medium or high educational needs: 1) transitional care when changing residential settings or post-hospitalization; 2) assisting family caregivers cope with caregiving responsibilities; 3) incorporating community-based services into care plans; and 4) and managing frail older adults. Social workers noted higher need than the other respondents for: 1) managing chronic pain; 2) managing care of patients with multiple chronic conditions; 3) having serious illness conversations; 4) diagnosing dementia; and 5) discussing advance care planning. Both social worker and other respondents indicated interactive case-based webinars; published tools, toolkits, tip sheets; and didactic webinars as their top three learning formats.

